# Ningxin-Tongyu-Zishen formula alleviates the senescence of granulosa cells on D-galactose-induced premature ovarian insufficiency mice

**DOI:** 10.18632/aging.205607

**Published:** 2024-02-28

**Authors:** Jia-Wen Ma, Zeng-Yan Xiong, Xing-Chu Cai, Xiang Li, Shi-Yan Ren, Shuai-Qi An, Zai-Yang Zhang, Yi-Zhou Zhang

**Affiliations:** 1 School of Basic Medical Sciences, Zhejiang Chinese Medical University, Hangzhou, China; 2 Zhejiang Famous Chinese Medicine Clinic, The Third Affiliated Hospital of Zhejiang Chinese Medical University, Hangzhou, China

**Keywords:** Ningxin-Tongyu-Zishen formula, premature ovarian insufficiency, ovarian granulosa cells, cell senescence, Sirt1/p53

## Abstract

Ningxin-Tongyu-Zishen formula (NTZF) is a clinical experience formula for the treatment of premature ovarian insufficiency (POI) in traditional Chinese medicine (TCM), and the potential mechanism is unknown. For *in vivo* experiments, POI mouse models (C57BL/6 mice), were constructed by subcutaneous injection of D-galactose (D-gal, 200 mg/kg). After treatment of NTZF (10.14, 20.27, 40.54 g/kg;) or estradiol valerate (0.15 mg/kg), ovarian function, oxidative stress (OS) and protein expression of Sirt1/p53 were evaluated. For *in vitro* experiments, H2O2 (200 μM) was used to treat KGN to construct ovarian granulosa cells (OGCs) cell senescence model. Pretreatment with NTZF (1.06 mg/mL) or p53 inhibitor (Pifithrin-α, 1 μM) was performed before induction of senescence, and further evaluated the cell senescence, OS, mRNA and protein expression of Sirt1/p53. *In vivo*, NTZF improved ovarian function, alleviated OS and Sirt1/p53 signaling abnormalities in POI mice. *In vitro* experiments showed that NTZF reduced the level of OS and alleviated the senescence of H2O2-induced KGN. In addition, NTZF activated the protein expression of Sirt1, inhibited the mRNA transcription and protein expression of p53 and p21. Alleviating OGCs senescence and protecting ovarian function through Sirt1/p53 is one of the potential mechanisms of NTZF in the treatment of POI.

## INTRODUCTION

Premature ovarian insufficiency (POI) is a complex refractory gynecological endocrine disease that occurs in women under 40 years old [1]. It is characterized by high gonadotropin levels and low estrogen levels, resulting in menstrual disorders, oligomenorrhea, infertility, and other clinical manifestations in patients with POI. Without timely intervention, POI will irreversibly and rapidly develop into premature ovarian failure (POF) and induce a range of complications including osteoporosis, genitourinary atrophy, neurodegenerative diseases, and cardiovascular diseases [2]. It has a great negative impact on the quality of life and the physical and mental health of patients [3]. A recent meta-analysis suggests that the prevalence of POI has risen to 3.5% [4]. At present, clinical therapy is mainly based on hormonal replacement therapy (HRT). But this method has a risk of promoting thrombosis, ovarian cancer, and breast cancer [5–7]. Although stem cell therapy has the potential to treat POI, there is a lack of evidence for large-scale clinical application [8, 9]. Therefore, how to treat POI more effectively and safely has become a great challenge for researchers.

As an important part of complementary medicine, traditional Chinese medicine (TCM) is increasingly used in the treatment of gynecological diseases [10, 11]. The project team has long been committed to the basic and clinical research of POI. We found that the medical history of POI patients has heart constraint symptoms caused by work or life, manifested as vexation, depression, wordlessness, difficulty falling asleep, and dreaminess [12]. Therefore, we believe that ‘heart constraint harassing kidney, consolidating the kidney and supplementing essence’ is the core etiology and pathogenesis of POI. According to the theory of ‘heart-kidney interaction’ in TCM. We modified Ningxin-Tongyu-Zishen Formula (NTZF) from Yijing Tang of *Fu Qingzhu Nv Ke* and Tianwang-Buxin-Dan of *Jiao Zhu Fu Ren Liang Fang*. The whole formula is composed of *Rehmanniae Radix Praeparata*, *Testudinis Carapax et Plastrum*, *Codonopsis Radix*, *Cuscutae Semen*, *Angelicae Sinensis Radix*, *Ziziphi Spinosae Semen*, *Moutan Cortex*, *Paeoniae Radix Alba*, *Dioscoreae Rhizoma* and *Bupleuri Radix*. It has the effects of nourish heart and tranquilize mind, tonify kidney and supplement essence. Retrospective clinical studies have shown that NTZF has a clear effect on the treatment of POI [12]. Therefore, we further explored its unclear treatment mechanism.

The etiology of POI is highly heterogeneous, and the pathogenesis is complex and unclear. Growing evidence suggests that the main reason of POI lies in the defects of follicular growth and development [13]. At the same time, because ovarian granulosa cells (OGCs) are important auxiliary cells that support follicular growth and maturation, they are closely related to ovarian function. Relevant studies have shown that OGCs senescence may be an important factor leading to POI [14]. Oxidative stress (OS) is considered to be one of the main causes of OGCs senescence [15]. Among them, reactive oxygen species (ROS) is one of the most important physiological inducers of cell senescence [16]. Although ROS are essential for cell growth and metabolism, when their content exceeds the body’s ability to neutralize, an imbalance between ROS and antioxidants occurs, accumulating in cells, leading to varying degrees of OS in DNA, lipids, and proteins, eventually causing cell senescence. Therefore, regulating OS is an important entry point for the treatment of POI. As a nicotinamide adenine dinucleotide-dependent deacetylase, Sirt1 can regulate a wide range of physiological functions and is the main regulator of metabolism and anti-oxidative stress [17]. It has been reported that Sirt1 is expressed in OGCs of various species, and activation of Sirt1 helps to improve ovarian function [14]. As one of the most critical pro-apoptotic genes, p53 can initiate various cellular responses including cell cycle arrest, cell apoptosis, and cell senescence in stress response [18]. Therefore, as a transcription factor regulated by Sirt1, inhibition of p53 expression is crucial in regulating and alleviating OGCs senescence [19]. In summary, this project further studied whether the Sirt1/p53 signaling pathway is involved in the process of NTZF treatment of POI.

In this study, we used the D-galactose (D-gal)-induced POI mouse model to verify the efficacy of NTZF in improving ovarian function. On this basis, NTZF was further used to intervene in H_2_O_2_-induced KGN cells. The aim of this study is to explore the role of NTZF in alleviating OGCs senescence and protecting ovarian function by alleviating OS in cells. And clarify the correlation between this effect and the Sirt1/p53 signaling pathway. We hope to provide more scientific basis for the clinical application of NTZF through this study.

## RESULTS

### Chemical composition analysis of NTZF

**Figure 1:**
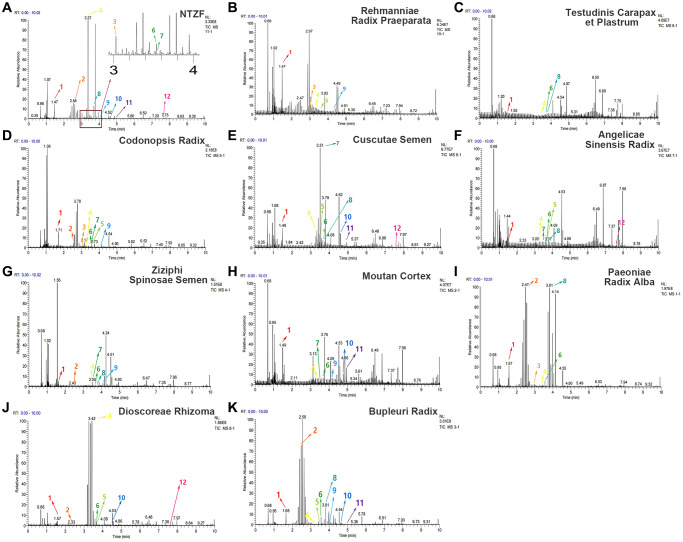
**The experimental scheme and LC-MS/MSTIC of NTZF.** (**A**) TIC of NTZF; (**B**–**K**) TIC of each Chinese medicinals in NTZF. Other labels: (No. 1) Adenosine, (No. 2) Gallic acid, (No. 3) 5-Hydroxymethyl-2-furaldehyde+, (No. 4) Magnoflorine, (No. 5) Caffeic acid, (No. 6) (+)-Catechin, (No. 7) Chlorogenic acid, (No. 8) Paeoniflorin, (No. 9) Ferulic acid, (No. 10) Quercetin, (No. 11) Isorhamnetin, and (No. 12) Linoleic acid.

NTZF is composed of ten kinds of medicinal materials (Table 1), and its chemical composition is complex. Figure 1A shows total ion chromatography (TIC) of NTZF, and the TIC of single medicinal material is shown in Figure 1B–1K. The compounds contained in NTZF and single medicinal material are marked in the figure, including Adenosine (No. 1), Gallic acid (No. 2), 5-Hydroxymethyl-2-furaldehyde+ (No. 3), Magnoflorine (No. 4), Caffeic acid (No. 5), (+)-Catechin (No. 6), Chlorogenic acid (No. 7), Paeoniflorin (No. 8), Ferulic acid (No. 9), Quercetin (No. 10), Isorhamnetin (No. 11), and Linoleic acid (No. 12). The content of different compounds in NTZF was determined by single point external standard method, which were calculated according to the peak area ratio of standard substance to target substance in TIC (Table 2). The 2D structure of each compound was found in https://pubchem.ncbi.nlm.nih.gov/. The contents of different compounds in the formula of medicinal materials are shown in Supplementary Tables 1-1 and 1-2.

**Table 1:** The composition of NTZF.

**English name**	**Chinese name**	**Plant or animal parts used**	**Crude herbs (g)**	**Voucher number**
*Rehmanniae Radix Praeparata*	Shu Di Huang	Root	30	20220104
*Testudinis Carapax et Plastrum*	Gui Jia	Carapace and plastron	12	20211124
*Codonopsis Radix*	Dang Shen	Root	12	20211127
*Cuscutae Semen*	Tu Si Zi	Seed	12	20210902
*Angelicae Sinensis Radix*	Dang Gui	Root	15	20220214
*Ziziphi Spinosae Semen*	Suan Zao Ren	Seed	12	20220214
*Moutan Cortex*	Mu Dan Pi	Root and bark	9	20211217
*Paeoniae Radix Alba*	Bai Shao	Root	12	20210501
*Dioscoreae Rhizoma*	Shan Yao	Root and stem	15	20211203
*Bupleuri Radix*	Chai Hu	Root	6	20211001

**Table 2:** The compounds in NTZF were identified by Q-Orbitrap LC-MS/MS.

**Peak No.**	**Compound**	**Molecular formula**	**CAS**	**Content (μg/mL)**	**Chemical structure**
1	Adenosine	C_10_H_13_N_5_O_4_	58-61-7	4.84	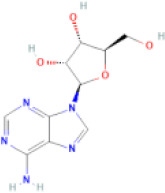
2	Gallic acid	C_7_H_6_O_5_	149-91-7	9.55	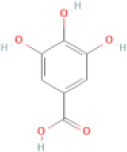
3	5-Hydroxymethyl-2-furaldehyde+	C_6_H_6_O_3_	67-47-0	7.68	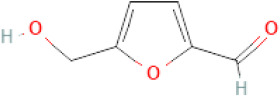
4	Magnoflorine	C_20_H_24_NO_4_^+^	2141-09-5	4.16	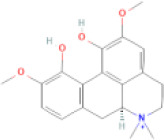
5	Caffeic acid	C_9_H_8_O_4_	331-39-5	3.18	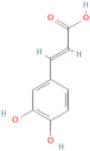
6	(+)-Catechin	C_15_H_14_O_6_	154-23-4	0.76	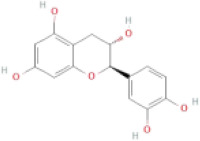
7	Chlorogenic acid	C_16_H_18_O_9_	327-97-9	0.52	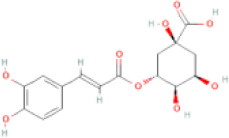
8	Paeoniflorin	C_23_H_28_O_11_	23180-57-6	150.86	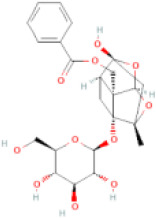
9	Ferulic acid	C_10_H_10_O_5_	1135-24-6	0.93	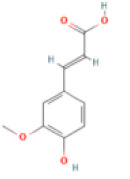
10	Quercetin	C_15_H_10_O_7_	117-39-5	0.35	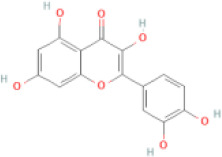
11	Isorhamnetin	C_16_H_12_O_7_	480-19-3	0.07	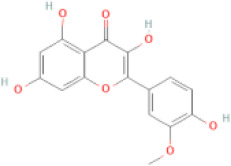
12	Linoleic acid	C_18_H_32_O_2_	60-33-3	2.11	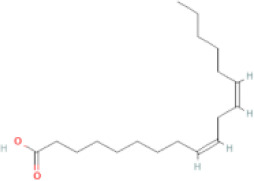

### NTZF effectively improved the ovarian function of POI mice

**Figure 2:**
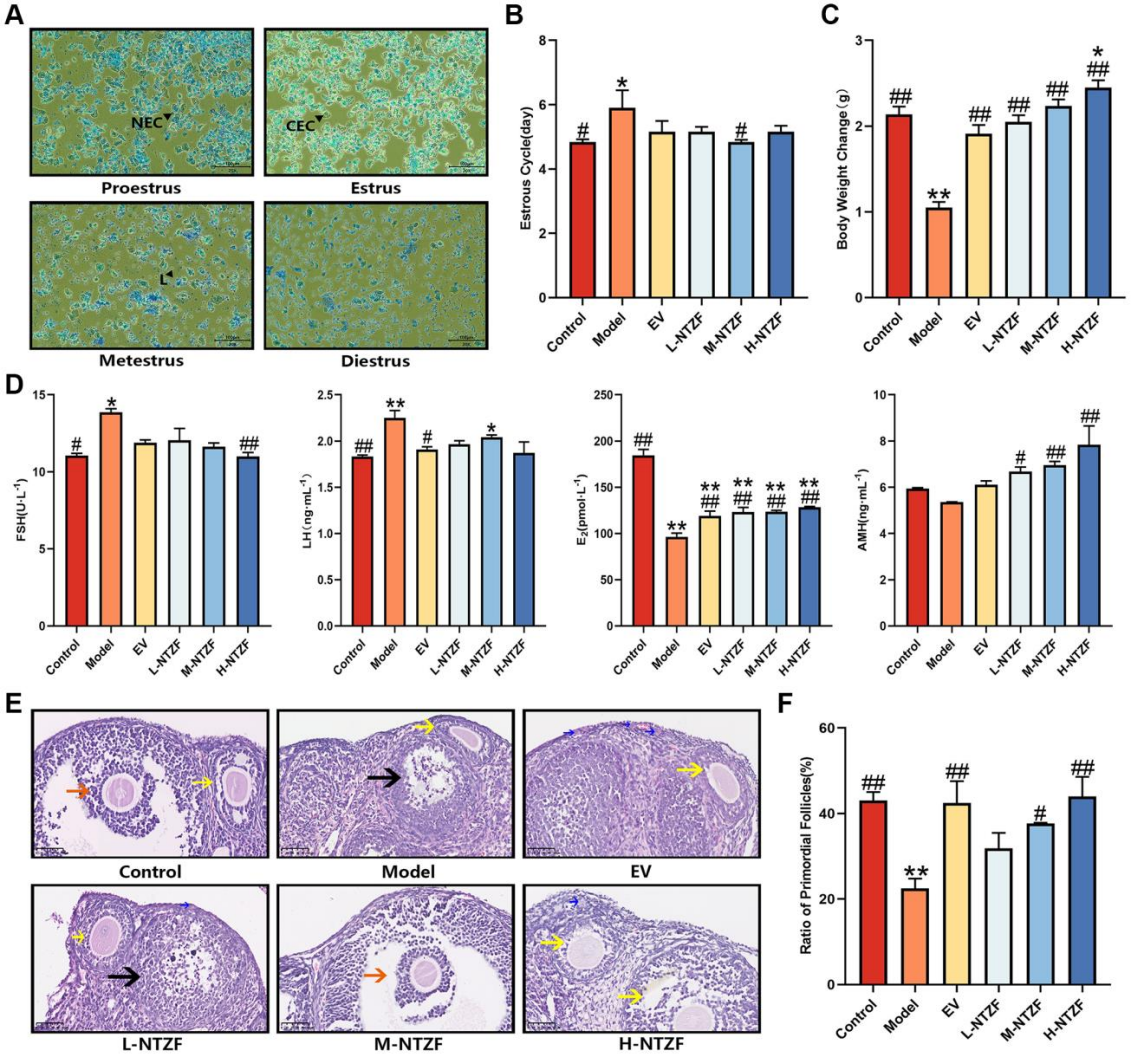
**NTZF can improve the estrous cycle, body weight, serum sex hormone levels and ovarian primordial follicle differentiation.** (**A**) Representative pictures of different estrous cycles (×200 magnification). NEC indicates nucleated epithelial cells, CEC indicates cornified epithelial cells, L indicates leukocytes. (**B**) Statistical figure of estrous cycle, *n* = 8. (**C**) Statistical chart of the difference in body weight of mice before and at the end of the experiment, *n* = 8. (**D**) The concentration changes of FSH, LH, E2 and AMH in serum of mice, *n* = 4. (**E**) Representative images of HE staining (×400 magnification). Blue arrows indicate primordial follicles, yellow arrows indicate growing follicles, orange arrow indicates mature follicles, and black arrow indicates atretic follicles. (**F**) Statistics of the ratio of primordial follicles in the ovary of mice. The ratio of primordial follicles = the number of primordial follicles/the total number of follicles ×100%, *n* = 3. (^*^p < 0.05, ^**^p < 0.01 versus control group; ^#^*p* < 0.05, ^##^*p* < 0.01, versus model group).

Vaginal smears were used to evaluate the estrous cycle of experimental animals. The estrous cycle is divided into four stages [20], including proestrus, estrus, metestrus, and diestrus. Proestrus is dominated by nucleated epithelial cells (NEC); cornified epithelial cells (CEC) are the main components of estrus; epithelial cells, cornified epithelial cells, and leukocytes (L) can be observed in metestrus; the diestrus is marked by leukocytes (Figure 2A). Compared with control group, the estrous cycle of mice in model group was significantly prolonged (^*^p < 0.05, Figure 2B), while M-NTZF could effectively restore the abnormal estrous cycle of mice (^#^*p* < 0.05, Figure 2B). The body weight of the mice in each group was compared before and at the end of the experiment. The body weight of the model mice increased slowly, which was significantly different from control group (^**^p < 0.01, Figure 2C). However, NTZF could effectively restore the weight gain of mice (^##^*p* < 0.01, Figure 2C). We speculated that this result may be due to the overall conditioning characteristics of TCM formula.

The levels of FSH, LH, E_2_, and AMH in the serum of mice were detected by ELISA (Figure 2D). Compared with control group, the levels of FSH (^*^p < 0.05) and LH (^**^p < 0.01) in model group were significantly increased, and E_2_ was significantly inhibited (^**^p < 0.01). Compared with model group, E_2_ was significantly increased after NTZF or EV treatment (^##^*p* < 0.01). At the same time, FSH of mice in H-NTZF group was significantly decreased (^##^*p* < 0.01), and LH of mice in estradiol valerate (EV) group was relatively decreased (^#^*p* < 0.05). In addition, AMH of mice in NTZF group was significantly higher than that in model group (^##^*p* < 0.01) and showed a gradient upward trend with the increase of NTZF treatment concentration. The above results suggested that NTZF improves the disordered serum sex hormone levels in POI mice.

HE staining was used to observe the morphological changes of follicles in the ovaries of mice. The typical HE staining images of follicles in different treatment groups were shown in Figure 2E. It was known that the main defect caused by POI is the decrease in the ratio of primordial follicle differentiation [21]. To study the improvement effect of NTZF on this situation, we compared the ratio of primordial follicles in ovarian sections (Figure 2F). We found that the ratio of primordial follicles in model group was significantly lower than that in control group (^**^p < 0.01). M-NTZF significantly increased the ratio of primordial follicles in POI mice (^#^*p* < 0.05), but EV and H-NTZF had a more obvious effect on increasing the ratio of primordial follicles in POI mice (^##^*p* < 0.01). The above results indicated that NTZF can improve the abnormal differentiation of primordial follicles caused by POI.

### NTZF increased the antioxidant capacity of ovaries in POI mice

**Figure 3:**
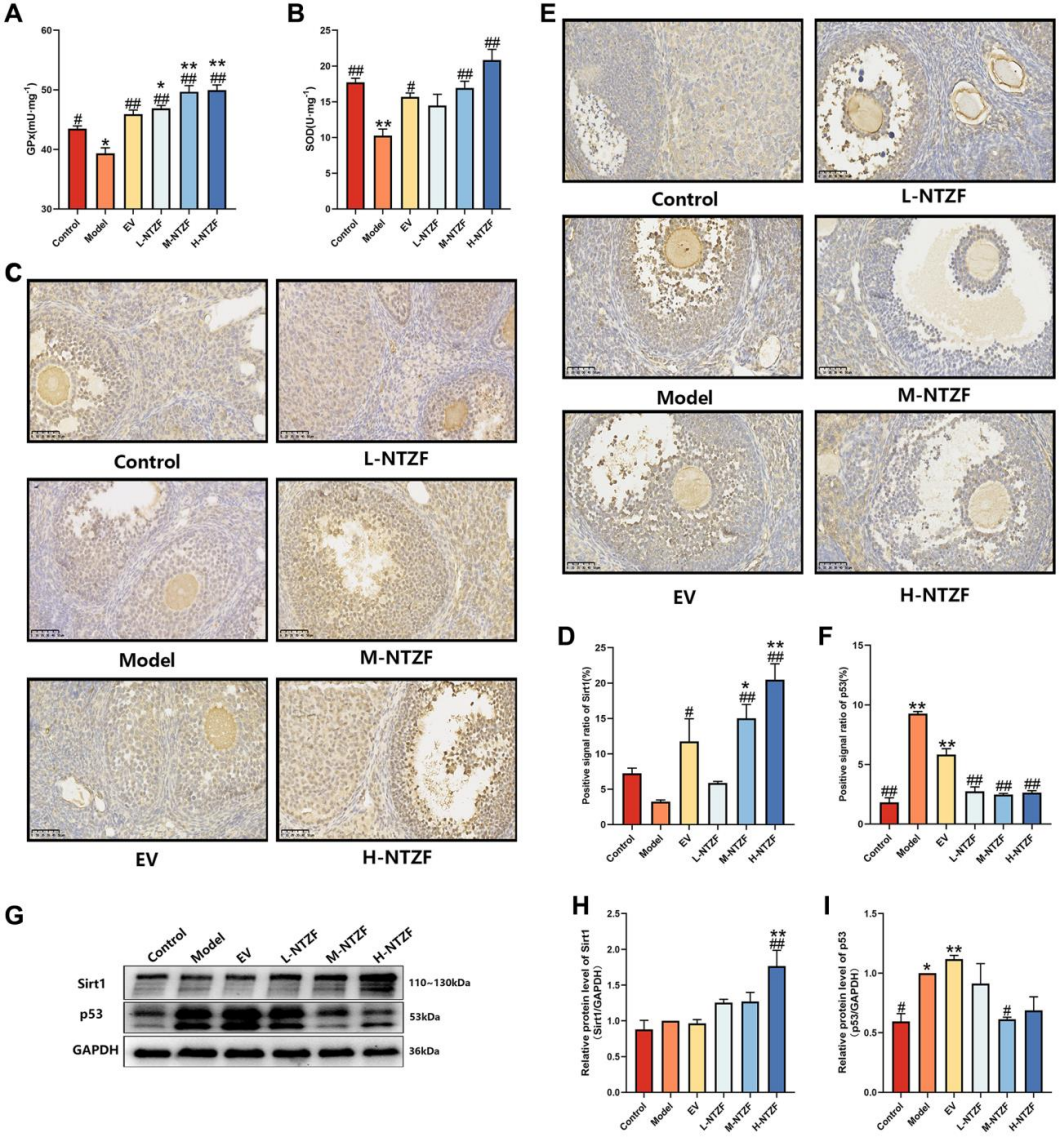
**NTZF alleviated oxidative stress and abnormal Sirt1/p53 signaling pathway in POI mice.** (**A**) The level of GPX in mouse ovary. (**B**) The level of SOD in mouse ovary. (**C**, **D**) The levels of Sirt1 in the ovaries of mice were detected by IHC (×400 magnification). (**E**, **F**) The levels of p53 in the ovaries of mice were detected by IHC (×400 magnification). (**G**–**I**) The protein expression levels of Sirt1 and p53 in mouse ovaries were detected by western blot. (^*^p < 0.05, ^**^p < 0.01, ^***^p < 0.001, versus control group, *n* = 3; ^#^*p* < 0.05, ^##^*p* < 0.01, ^###^*p* < 0.001, versus model group, *n* =3).

To evaluate the effect of NTZF on OS in the ovarian tissue of POI mice, we measured the levels of GPX and SOD (Figure 3A, 3B). Compared with control group, GPX (^*^p < 0.05) and SOD (^**^p < 0.01) in model group were significantly decreased. After EV and NTZF treatment, the decrease of GPX can be effectively reversed (^##^*p* < 0.01). After NTZF treatment, the level of GPX in mice was significantly higher than that in control group (^*^p < 0.05). At the same time, EV, M-NTZF, and H-NTZF treatment could up-regulate the level of SOD in mouse ovarian tissue (^#^*p* < 0.05), and the ability of M-NTZF and H-NTZF to increase the level of SOD was particularly significant (^##^*p* < 0.01). These results suggest that NTZF can play a therapeutic effect on POI by improving the antioxidant capacity of mouse ovaries.

Because Sirt1/p53 signaling pathway plays an important role in regulating OS [22], we used IHC and western blot to evaluate the expression of Sirt1 and p53 in mouse ovarian tissue (Figure 3C, 3E, 3G). Compared with control group, there was no difference in the expression of Sirt1 in the ovary of model group, but H-NTZF treatment could significantly increase the expression of Sirt1 (^##^*p* < 0.01, Figure 3D, 3H). In the ovaries of mice in model group, both IHC and western blot showed that the expression of p53 was significantly up-regulated compared with control group (^*^p < 0.05, Figure 3F, 3I). Although IHC results suggested that all concentrations of NTZF could significantly reverse the up-regulation of p53 (^##^*p* < 0.01, Figure 3F), western blot showed that only M-NTZF significantly inhibited the protein expression of p53 in mouse ovaries (^#^*p* < 0.05, Figure 3I). These results indicate that NTZF can restore the proliferation of OGCs in the ovaries of POI mice by influencing Sirt1/p53 signaling pathway.

### NTZF alleviated OGCs senescence and improved antioxidant capacity

**Figure 4:**
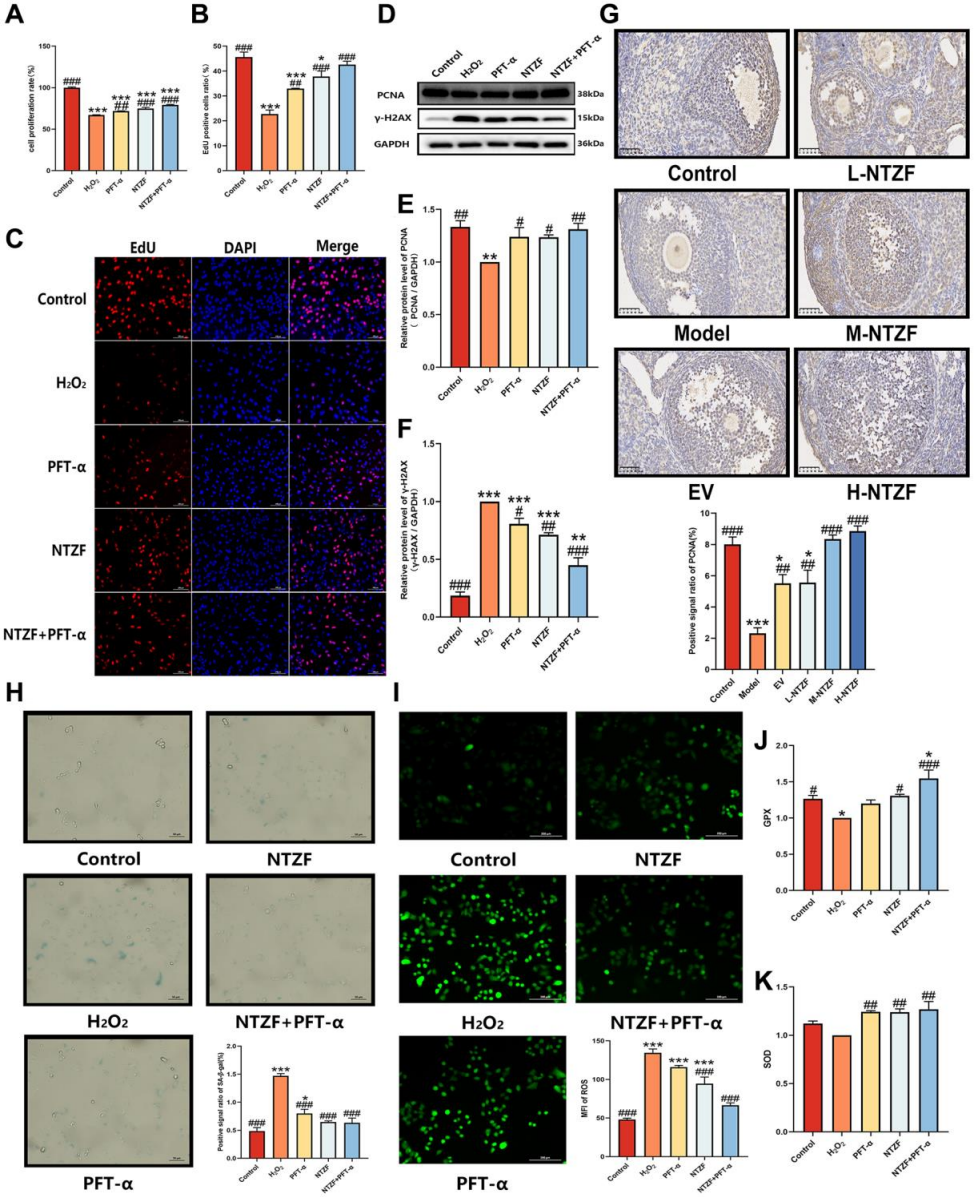
**NTZF alleviated OGCs senescence and improved antioxidant capacity.** (**A**) The cell proliferation rate was detected by CCK-8. (**B**, **C**) Cell proliferation was detected by EdU cell proliferation staining (×200 magnification). The ratio of EdU positive cells = the number of EdU stained cells/the number of DAPI stained cells ×100%. (**D**–**F**) The protein expression of PCNA and γ-H2AX was detected by western blot. (**G**) The levels of PCNA in the ovaries of mice were detected by IHC (×400 magnification). (**H**) SA-β-gal staining was used to detect cell senescence (×400 magnification). Blue staining indicated cell senescence. (**I**) The intracellular ROS level was detected by DCFH-DA probe. Green fluorescence indicated ROS expression. (**J**) The level of intracellular GPX. (**K**) The level of intracellular SOD content (^*^p < 0.05, ^**^p < 0.01, ^***^p < 0.001, versus control group, *n* = 3; ^#^*p* < 0.05, ^##^*p* < 0.01, ^###^*p* < 0.001, versus H_2_O_2_ group, *n* = 3).

To further explore whether NTZF could alleviate the senescence of OGCs through antioxidant effect to protect the ovaries of POI patients, we selected KGN cells for following research. In the preliminary experiment, we screened the optimum concentration of H_2_O_2_ (200 μM) and NTZF (1.06 mg/mL) to treat cells with CCK-8 and crystal violet staining (Supplementary Figure 1). According to the results of CCK-8 (Figure 4A) and EdU cell proliferation staining (Figure 4B, 4C), the cell proliferation rate of H_2_O_2_ group was significantly lower than that of control group (^***^p < 0.001). Compared with H_2_O_2_ group, the proliferation rate of cells treated with p53 inhibitor—PFT-α was significantly increased (^##^*p* < 0.01), while the proliferation rate of NTZF group and NTZF+PFT-α group was significantly increased (^###^*p* < 0.001). At the same time, western blot results also suggested that the proliferation-related index PCNA also had higher protein expression in NTZF group and NTZF+PFT-α group (^#^*p* < 0.05, Figure 4D, 4E). For *in vivo* experiments, IHC results of PCNA also showed that compared with control group, the expression of PCNA in the ovary of POI model group was significantly decreased (^***^p < 0.001, Figure 4G), while EV and NTZF treatment promoted the expression of PCNA in the ovary (^##^*p* < 0.01). The above results suggest that NTZF can increase the level of PCNA. In addition, we quantified the protein expression of γ-H2AX, a marker of DNA damage. The results showed that the cells in each group treated with H_2_O_2_ showed obvious DNA damage (Figure 4D, 4F). However, the expression of γ-H2AX protein in NTZF group and NTZF+PFT-α group was significantly lower than that in H_2_O_2_ group (^##^*p* < 0.01), indicating that NTZF has a protective effect on H_2_O_2_-induced DNA damage in KGN cells. It was known that proliferation inhibition and DNA damage were typical characteristics of cell senescence. To further clarify the cell senescence of KGN cells, we also stained the biomarker SA-β-gal of cell senescence (Figure 4H). Compared with H_2_O_2_ group, the ratio of positive signals in PFT-α group, NTZF group, and NTZF+PFT-α group was significantly decreased (^###^*p* < 0.001). In summary, NTZF had a significant protective effect on H_2_O_2_-induced KGN cells senescence.

To clarify the effect of NTZF on OS in OGCs, the levels of ROS, GPX, and SOD in KGN cells were detected again (Figure 4I–4K). The cells were labeled by DCFH-DA probe, and the results showed that the ROS level in KGN cells in H_2_O_2_ group was significantly increased (^***^p < 0.001), while the ROS level in KGN cells treated with NTZF or NTZF combined with PFT-α in advance was significantly lower than that in H_2_O_2_ group (^###^*p* < 0.001). At the same time, KGN cells pretreated with NTZF also showed a significant increase in GPX (^#^*p* < 0.05) and SOD (^##^*p* < 0.01). Therefore, NTZF improved the antioxidant capacity of ovarian granulosa cells. Therefore, NTZF can improve the antioxidant capacity of OGCs and correct the OS state of H_2_O_2_-induced imbalance between intracellular ROS and antioxidant levels.

### NTZF protects OGCs by regulating Sirt1/p53 signaling pathway

**Figure 5:**
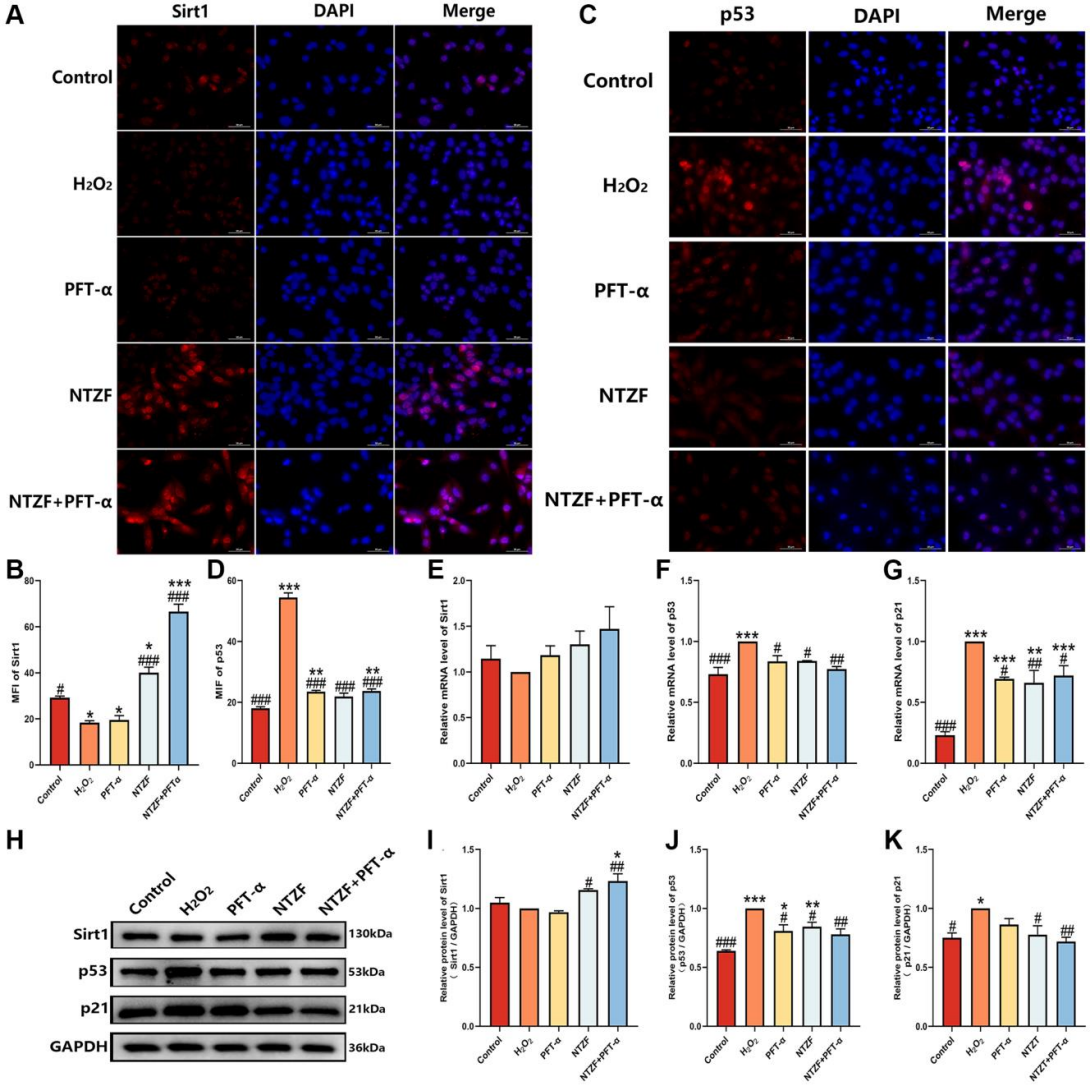
**NTZF protecting OGCs in vitro was associated with Sirt1/p53 signaling pathway.** (**A**–**D**) The expression of Sirt1 and p53 in each group was detected by IF (×400 magnification). (**E**–**G**) The relative mRNA levels of Sirt1, p53, and p21 were analyzed by q-PCR. (**H**–**K**) Western blot was used to analyze the protein expression of Sirt1, p53, and p21 in each group (^*^p < 0.05, ^**^p < 0.01, ^***^p < 0.001, versus control group, *n* = 3; ^#^*p* < 0.05, ^##^*p* < 0.01, ^###^*p* < 0.001, versus H_2_O_2_ group, *n* = 3).

In order to further clarify the mechanism of NTZF protecting H_2_O_2_-induced OGCs through Sirt1/p53 signaling pathway, we detected the mRNA and protein expression of Sirt1, p53 and p21 in cells. The expression of Sirt1 and p53 in each group of cells was semi-quantitatively determined by IF (Figure 5A–5D). The results showed that compared with control group, the expression of Sirt1 in H_2_O_2_ group was significantly decreased (^*^p < 0.05), and the expression of p53 was significantly increased (^***^p < 0.001). Compared with H_2_O_2_ group, the expression of Sirt1 in NTZF group and NTZF + PFT-α group was significantly increased (^###^*p* < 0.001), and the expression of p53 was significantly decreased (^###^*p* < 0.001). The expression levels of Sirt1, p53 and p21 mRNA were detected by q-PCR (Figure 5E–5G). It was found that there was no significant difference in the level of Sirt1 mRNA in each group (*p* > 0.05), but NTZF treatment could significantly inhibit the transcription of p53 mRNA (^#^*p* < 0.05) and p21 mRNA (^##^*p* < 0.01). In western blot detection (Figure 5H), the expression trend of Sirt1 protein in NTZF group and NTZF + PFT-α group was the same as that of IF (Figure 5I). The protein expression of p53 and p21 was significantly lower than that of H_2_O_2_ group (^#^*p* < 0.05, Figure 5J, 5K), and the inhibitory effect of NTZF + PFT-α group on p53 protein expression was more significant (^##^*p* < 0.01). In summary, NTZF can promote the expression of Sirt1 protein level, inhibit the transcription and protein expression of p53 and p21, and thus play a role in protecting OGCs.

## DISCUSSION

POI is a clinically difficult to treat gynecological disease that brings great physical and mental disturbance to women. Its etiology includes chromosomal abnormalities, gene mutations, autoimmune factors, iatrogenic factors, and so on. But majority etiologies of patients have undetermined etiology and are idiopathic. And its proportion is as high as 75–90% [23]. NTZF is a modified formula from the classical formula, which has a specific clinical effect [12]. It can not only improve the levels of FSH and E_2_ in patients with POI, but also restore the normal menstrual cycle. Furthermore, NTZF can also eliminate clinical symptoms such as vexation, depression, wordlessness, difficulty falling asleep, and dreaminess in patients. However, the specific mechanism of NTZF in the treatment of POI is unclear. This study found that NTZF can treat D-gal-induced POI mice, and its therapeutic mechanism may be to improve the antioxidant capacity and alleviate OGCs cell senescence by affecting the Sirt1/p53 signaling pathway. It provides a reliable scientific basis for the wide application of NTZF in clinic.

Subcutaneous injection of D-gal is one of the classic methods for aging model and is widely used in animal modeling of aging-related diseases [24]. At present, this method has been successfully applied to the construction of POI model and the study of POI disease, which is a relatively recognized and mature method for the construction of POI model [25]. Therefore, this method was selected to construct the POI model in this study. After 42 days of subcutaneous injection of D-gal, we detected that the estrous cycle of model group was prolonged, the level of FSH was increased, the level of E_2_ was reduced, and the ratio of primordial follicles was reduced. The above results suggest that the changes of sex hormone levels and pathological characteristics of ovarian tissue in the POI mouse model constructed by us are consistent with the performance of POI patients, indicating that the POI model has been successfully replicated in this experiment. After using NTZF to treat POI model, we found that NTZF can shorten the estrous cycle of POI mice, reduce the levels of FSH and LH, increase the levels of E_2_ and AMH, and increase the ratio of primordial follicles. These results preliminarily demonstrate the effectiveness of NTZF in treating POI.

NTZF is a TCM formula with complex chemical components. This study identified 12 compounds in NTZF (Table 2, Figure 1B). This is not only of great significance for the quality control of NTZF, but also lays a foundation for further exploration of the pharmacological effects of NTZF in the future. Because TCM has the characteristics of multi-component, multi-target and coordinated integration. Therefore, it is difficult to determine which chemical component of NTZF plays a major role in the treatment of POI. According to reports, interestingly, 12 identified components showed antioxidant activity. For instance, Magnoflorine can reduce the production of ROS in Aβ-induced PC12 cells [26]. The role of Paeoniflorin in acute liver injury is to reduce the levels of liver ROS and MDA, and increase SOD activity [27]. In addition, Gallic acid [28], Quercetin [29], Isorhamnetin [30] have been shown to enhance the activity of ovarian antioxidant enzymes and regulate the level of OS. The above findings inspire us that NTZF may play a role in the treatment of POI by improving ovarian antioxidant capacity. In the study, we also found that the levels of GPX and SOD in the ovaries of POI mice were significantly increased after NTZF treatment. This suggests that NTZF can protect ovarian function by improving ovarian antioxidant capacity, which provides valuable clues for subsequent mechanism research.

Reproductive ability is highly sensitive to redox homeostasis and oxidase levels. Studies have shown that the accumulation of ROS above normal levels, thereby inducing OGCs senescence, is one of the important pathogenic factors leading to POI. The increase of ROS and the decrease of antioxidant defense will lead to oxidative damage of cell structure, lipid peroxidation, enzyme inactivation, protein oxidation and DNA damage. According to the ‘free radical theory’, ROS is the driving force of cell senescence, and senescence is the result of oxidative damage. In this study, H_2_O_2_-induced KGN cells showed an increase in ROS and a decrease in antioxidant enzymes such as GPX and SOD, indicating that OS does exist in cells. The results of CCK-8, EdU cell proliferation staining and PCNA protein quantification suggested that H_2_O_2_-induced KGN cells showed significant proliferation inhibition. In addition, the expression of DNA damage index γ-H2AX protein increased, and the positive area of SA-β-gal staining increased, further indicating the existence of cell senescence. It can be seen that the imbalance between ROS and antioxidant enzymes indeed induces the senescence of OGCs. After NTZF treatment, intracellular GPX and SOD increased, ROS decreased, cell proliferation activity increased significantly, and DNA damage decreased significantly. These results demonstrate that NTZF can improve the antioxidant capacity of OGCs, which is consistent with the results of *in vivo* experiments. The above results also suggest that the increase of antioxidant capacity contributes to the alleviation of OGCs senescence.

As a NAD^+^-dependent histone deacetylase closely related to cell differentiation, senescence and energy metabolism, Sirt1 is a sensor of cell redox state. Studies have suggested that activation of Sirt1 can effectively improve damaged ovarian function under OS [31]. And p53 is the redox target molecule of Sirt1. Sirt1 can inhibit p53-dependent transcription by promoting p53 deacetylation [32]. p53 is a transcription factor that mediates genome damage-induced senescence. Studies have shown that telomere DNA deletion [33], ionizing radiation [34], chemotherapy drugs [35] and other factors can trigger p53-mediated senescence process. As one of the transcriptional targets of p53, p21 is also a gene that regulates cell proliferation and cell senescence [36]. Moreover, p53 and p21 are often used as markers of cell senescence in many aging-related studies. In this study, we found that although NTZF did not significantly activate the transcription of Sirt1 mRNA, it activated its protein expression. This indicates that the effect of NTZF on Sirt1 is mainly at the protein level, not at the transcriptional level. NTZF significantly inhibited the mRNA and protein expression of p53 and p21 by activating Sirt1. Therefore, combined with the above research, it is correct to speculate that NTZF alleviates cell senescence through Sirt1/p53 signaling pathway. In addition, we increased the treatment of KGN cells by PFT-α in the experiment. It is known that PFT-α is an inhibitor of p53 and can inhibit p53-dependent gene transcription [37]. In our study, we found that the mRNA and protein expression of p53 and p21 in NTZF-treated cells was similar to that in PFT-α-treated cells. Interestingly, their cell proliferation and cell senescence indicators are quite different, and the expression of Sirt1 protein also has a greater tendency to separate. NTZF group showed a tendency to be superior to the PFT-α group. This may be related to the multi-component and multi-target characteristics of NTZF as a TCM formula. Sirt1/p53 signaling pathway is only one of the signaling pathways to protect the ovary. It is precisely for this reason that when NTZF and PFT-α are combined, the effect on Sirt1/p53 signaling pathway is more significant, indicating that there is a synergistic effect between NTZF and PFT-α. In summary, alleviating OGCs senescence and protecting ovarian function through Sirt1/p53 signaling pathway is one of the potential mechanisms of NTZF in the treatment of POI.

For *in vitro* and *in vivo* experiments, this study identified the therapeutic effect of NTZF in alleviating the senescence of granulosa cells and protecting ovarian function. Moreover, the mechanism by which NTZF exerts therapeutic effects through the Sirt1/p53 signaling pathway has been preliminarily explored. To further validate the therapeutic mechanism of NTZF, a lentiviral transfection approach will be used in future studies to clarify the role of NTZF in this signaling pathway by silencing p53. Furthermore, based on the fact that Sirt1 is only affected in protein expression, our team will explore the changes in Sirt1 protein modification under the action of NTZF. We hope to provide scientific basis for the clinical application of NTZF by delving deeper into its role in regulating the Sirt1/p53 signaling pathway.

## CONCLUSIONS

In this study, D-gal-induced POI mouse model was used to evaluate the efficacy of NTZF in the treatment of POI, and H_2_O_2_-induced KGN cells were used to explore the mechanism of NTZF in the treatment of POI. Our results suggest that NTZF can enhance the antioxidant capacity of OGCs and alleviate cell senescence, thereby improving ovarian function. This effect is related to this mechanism, which is that NTZF activates Sirt1 and inhibits p53. In summary, these findings provide a theoretical and experimental basis for the application of NTZF in the clinical treatment of POI patients.

## MATERIALS AND METHODS

### Preparation of drugs

NTZF is composed of ten kinds of medicinal materials, including Rehmanniae Radix Praeparata, Testudinis Carapax et Plastrum, Codonopsis Radix, Cuscutae Semen, Angelicae Sinensis Radix, Ziziphi Spinosae Semen, Moutan Cortex, Paeoniae Radix Alba, Dioscoreae Rhizoma, and Bupleuri Radix (in a weight ratio of 10:4:4:4:5:4:3:4:5:2, Table 1). All medicinal materials were purchased from the Third Affiliated Hospital of Zhejiang Chinese Medicine University. NTZF was prepared as follows [38]: the medicinal materials were soaked in distilled H_2_O (dH_2_O, 1:8 w/v) for 30 min, and then boiled with strong fire until the water boiled. Subsequently, the medicinal materials were decocted with soft fire for 40 min, and then the water decoction was filtered out. The medicinal materials were decocted again in the same way. After mixing, the decoction was concentrated (2.7 g/mL). The liquid was stored at 4°C.

In this experiment, we also chose EV tablets (commonly used drugs for POI in clinic, J20171038, Bayer, Germany) as a positive control, and EV suspension (0.01 mg/mL) was diluted with dH_2_O.

### Chemical composition analysis of NTZF

We used Q-Orbitrap liquid chromatography-mass spectrometry/mass spectrometry (LC-MS/MS) to qualitatively analyze NTZF, and a total of 71 chemical components were detected in previous studies [38]. In this study, we further quantified 12 of the 71 chemical components contained in NTZF. In addition, the contents of these 12 chemical components in single medicinal materials were also detected. The decoction was extracted using anhydrous methanol in a ratio of 5:1, and the resulting extract was utilized for subsequent analysis. The MS conditions were as follows: Ion source: electrospray ionization source (ESI); Scanning mode: positive and negative ion switching scanning; Detection methods: parallel reaction monitoring (PRM); Resolution: 17500; Scan range: 100-1000 m/z; Spary Voltage: 3.2 kV (Pos, Neg); Capillary Temperature: 300°C; Collision gas: high purity argon (purity≥99.999%); Sheath gas: nitrogen (purity≥99.999%), 40 Arb; Auxiliary gas: nitrogen (purity≥99.999%), 15 Arb, 350°C; Data acquisition time: 10.0 min. The chromatographic conditions were as follows: Chromatographic column: AQ-C18 150 × 2.1 mm, 1.8 μm, Welch; Flow rate: 0.3 mL/min; Needle washing liquid: methanol; Column oven temperature: 35°C; Automatic sampler temperature: 10°C; Automatic sampler injection volume: 5 μL; Water phase: 0.1% formic acid/water; Organic phase: 0.1% formic acid/acetonitrile. The gradient elution process of mobile phase is shown in Table 3. Reference standards, including Adenosine (B21356), Gallic acid (B20851), 5-Hydroxymethyl-2-furaldehyde+ (B21832), Magnoflorine (B20882), Caffeic acid (B20660), (+) -Catechin (B21722), Chlorogenic acid (B20782), Paeoniflorin (B21148). Ferulic acid (B20007), Quercetin (B20527), Isorhamnetin (B21554), Linoleic acid (B21421), were purchased from Shanghai Yuanye Bio-Technology Co., Ltd., (China).

**Table 3:** Gradient elution process of mobile phase.

**Times (min)**	**0.1% Formic acid/Water (%)**	**0.1% Formic acid/Acetonitrile (%)**
0	95	5
0.5	95	5
5.0	5	95
8.0	5	95
8.5	95	5
10	95	5

### Animals and treatment

Female C57BL/6 mice aged 6–8 weeks weighing 16–18 g, were purchased from Zhejiang Academy of Medical Sciences (China). The mice were routinely raised in Zhejiang Chinese Medical University Laboratory Animal Research Center. This is a specific pathogen-free (SPF) environment with 12 h light-dark cycles. Its ambient temperature is maintained at 20~25°C, and relative humidity is maintained at 40~70%.

**Figure 6:**
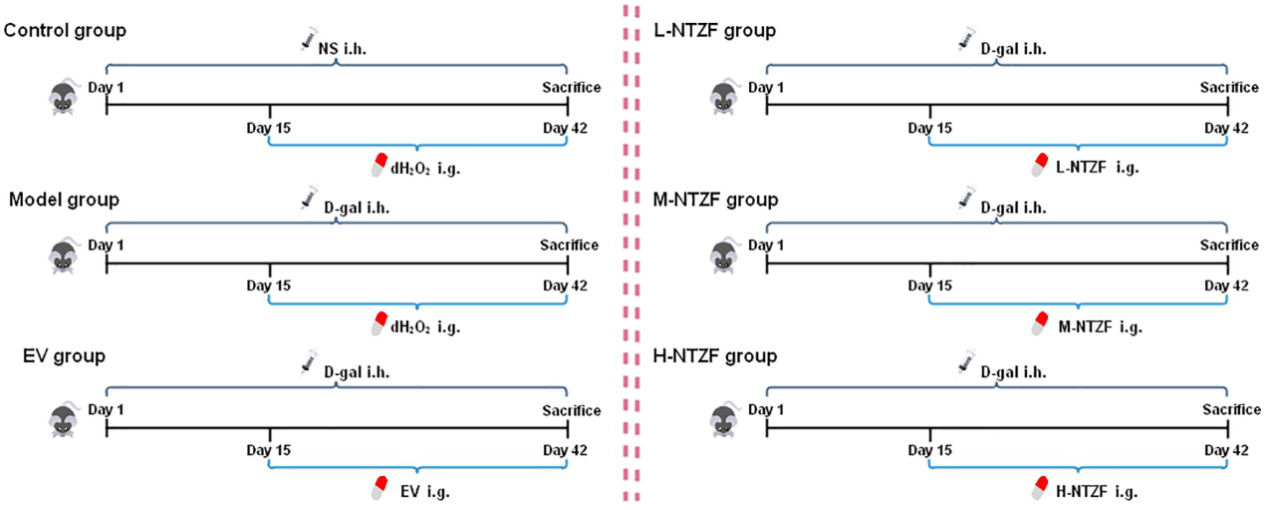
Overview of experimental scheme.

Before the experiment, mice were acclimated for one week. These mice were randomly divided into six groups, including Control group, Model group, EV group (0.15 mg/kg), L-NTZF group (10.14 g/kg), M-NTZF group (20.27 g/kg) and H-NTZF group (40.54 g/kg). Except for the Control group, the other groups were subcutaneously injected with D-gal (200 mg/kg) to establish a POI mouse model [39]. The mice in control group were injected with an equal volume of normal saline (NS). Subcutaneous injection lasted from the first day of the experiment to the day of sacrifice for 42 days, and the day of subcutaneous injection was defined as Day1 (Figure 6). The clinical therapeutic dose of NTZF for women weighing 60 kg was 135 g/day, and the clinical therapeutic dose of EV tablets was 1 mg/day. The dose used in mice was calculated by the recommended human-mice conversion ratio (the ratio of human to mice was 1:9). In the study, the mice in the L-NTZF group were given 1/2 times the clinical equivalent dose, the mice in the M-NTZF group were given the same dose as the clinical equivalent dose, and the mice in the H-NTZF group were given 2 times the clinical equivalent dose. From Day15, each group began to give medicine intervention, and the dosage and duration of each group were shown in Table 4. On Day42, mice were anesthetized by intraperitoneal injection of 1% pentobarbital sodium solution (50 mg/kg), and blood and ovaries were collected. At the end of sampling, the mice were sacrificed by excessive anesthesia.

**Table 4:** Grouping and treatment of experimental animals.

**Groups**	**Administration**	**Numbers of mouse**	**Dose (mL/kg)**	**Duration (days)**
Control	dH_2_O_2_	8	15	28
Model	dH_2_O_2_	8	15	28
EV	Estradiol Valerate suspensoid	8	15	28
L-NTZF	The water extract of NTZF	8	15	28
M-NTZF	The water extract of NTZF	8	15	28
H-NTZF	The water extract of NTZF	8	15	28

### Observation of estrous cycle

From Day28, the estrous cycle of mice was examined daily for 15 days, and the body weight of each mouse was monitored before and at the end of the experiment. Vaginal smear cytology was performed at 15 p.m. every day. The vaginal cavity was repeatedly washed with 10 μL of NS. Vaginal secretions were smeared on slides and fixed with 95% ethanol to obtain smears. The smears were stained with 0.23% Alkaline methylene blue staining solution (R24063, Yuanye, China). Finally, the morphology of vaginal exfoliated cells was observed by optical microscope with ×100 magnification. According to the observed ratio of different cells, the stage of estrous cycle in mice was determined.

### ELISA

ELISA was performed according to the manufacturer’s guidelines. The concentrations of FSH (MM-45654M1, Meimian, China), LH (MM-44039M1, Meimian, China), E_2_ (MM-0566M1, Meimian, China), and AMH (MM-44204M1, Meimian, China) in mice serum were determined using mice-specific ELISA kits.

### Ovarian follicle count and morphological observation

Mice ovarian tissues were fixed with 4% paraformaldehyde for at least 24 h. Then the fixed tissues were dehydrated with gradient concentration of alcohol, and embedded in paraffin. Mice ovarian wax blocks were made into 4 μm sections, baked at 60°C for 2 h, and then stained with HE. The digital pathological scanning system (Hamamatsu, Japan) was used to scan the pathological sections of mice ovarian tissue, and the number of follicles in ovarian sections was counted. Follicles can be divided into four stages [40] (Figure 2E): primordial follicles, growing follicles, mature follicles, and atresia follicles. Primordial follicles (blue arrows) were characterized by oogonia, which was surrounded by flat granulosa cells; growing follicles (yellow arrows) were characterized by one or more layers of cubic granulosa cells around the oocytes, and even follicular cavities; mature follicles (orange arrows) were characterized by a large follicular cavity with obvious cumulus; atresia follicles (black arrows) were characterized by unclear egg cell structure, shrunken zona pellucida, and collapsed follicle walls.

### Cell culture and grouping

The KGN human granulosa cell line (cat. no. iCell-h298, China) conforming to short tandem repeats identification was purchased from iCell Biotechnology Co., Ltd. (China). KGN cells were cultured in DMEM/F-12 containing 10% FBS. The incubator conditions were 37°C and 5% CO_2_. KGN cell in logarithmic growth phase was divided into control group, H_2_O_2_ group (200 μM H_2_O_2_) [41], PFT-α group (200 μM H_2_O_2_+1 μM PFT-α), NTZF group (200 μM H_2_O_2_ + 1.06 mg/mL NTZF), PFT-α + NTZF group (200 μM H_2_O_2_+1 μM PFT-α+1.06 mg/mL NTZF). NTZF diluted with PBS was added to NTZF group and PFT-α+NTZF group, and the same volume of PBS was added to the other groups. At the same time, PFT-α group and PFT-α+NTZF group were added with PFT-α dissolved in DMSO, and the other groups were added with equal volume of DMSO. After 24 h, H_2_O_2_ group, PFT-α group, NTZF group and PFT-α+NTZF group were added with 200 μM H_2_O_2_, and control group was added with the same volume of PBS. After 2 h of H_2_O_2_ intervention, cells in each group were subjected to subsequent detection.

### Cell proliferation analysis

Cell Counting Kit (CCK-8, C0039, Beyotime, China) was used to evaluate the proliferation of KGN cells. KGN cells were inoculated into 96-well plates at 8 × 10^3^ cells/well. After the intervention, 10 μL CCK-8 was added to each well and continued to incubate for 2.5 h. Finally, the OD value was measured at 450 nm using a multifunctional microplate reader (M200 pro, Tecan, Switzerland).

According to the manufacturer’s guidelines in BeyoClick™ EdU Cell Proliferation Kit with Alexa Fluor 555 (C0075S, Beyotime, China), KGN cells were inoculated into 24-well plates and cultured. After the intervention, the proliferating cells were labeled with EdU. Click reaction solution was prepared according to the instructions. After 30 min incubation in the dark, the nucleus was labeled with Antifade Mounting Medium with DAPI (MA0222, Meilunbio, China). Finally, three random field images were taken with a fluorescence inverted microscope (Axio Observer A1, Zeiss, Germany). ImageJ V.1.8.0 software was used to calculate the number of EdU-stained cells and DAPI-stained cells.

### SA-β-gal staining

The experiment was performed according to the guidelines in SA-β-Gal Staining Kit (C0602, Beyotime, China). To put it simply, we cultured KGN cells in 24-well plates. The cells were fixed with staining fixative at room temperature for 15 min, and then the staining solution was added and incubated at 37°C overnight. The next day, the staining results were observed under a microscope, and the percentage of senescent cells (blue stained cells) was counted using ImageJ V.1.8.0 software.

### Biochemical estimations of OS markers

According to the manufacturer’s guidelines in the Reactive Oxygen Species Assay Kit (E004-1-1, Njjcbio, China), KGN cells were inoculated into 6-well plates and cultured. After the intervention, the cells were labeled with 5 μM DCFH-DA probe. Images were taken using a fluorescence inverted microscope (Axio Observer A1, Zeiss, Germany). The mean fluorescence intensity (MFI) of ROS in the image was analyzed by ImageJ V.1.8.0 software, and the data were normalized.

Proteins in mouse ovarian tissue or KGN cells were extracted with sample preparation solution, and the protein concentration was detected by BCA Protein Assay Kit (P0012, Beyotime, China). According to the guidelines of Total Glutathione Peroxidase Assay Kit (S0059S, Beyotime, China), various reagents were sequentially added to 96-well plates and incubated at room temperature for 30 min. Then DTNB solution was added and incubated at room temperature for 10 min. A multifunctional microplate reader (M200 pro, Tecan, Switzerland) was used to determine the OD value at a wavelength of 412 nm, which was used to calculate the GPX content of the sample. According to the guidelines of Total Superoxide Dismutase Assay Kit (S0101M, Beyotime, China), various reagents were added to 96-well plates in order. After incubation in 96-well plates at 37°C for 30 min, the OD value was measured at 450 nm using a multifunctional microplate reader (M200 pro, Tecan, Switzerland) to calculate the SOD content of the sample.

### IHC staining

After dewaxing 4 μM mouse ovarian tissue sections, they were immersed in antigen repair solution and heated by microwave oven for antigen repair. Tissue sections were permeabilized with Enhanced Immunostaining Permeabilization Buffer (P0097, Beyotime, China) for 5 min, and then incubated with Enhanced Endogenous Peroxidase Blocking Buffer (PV-9000, ZSGB-BIO, China) for 10 min. At 37°C, the tissues were blocked with 5% goat serum for 30 min. The primary antibody was dropped and incubated overnight at 4°C. On the second day, the enhancer solution (PV-9000, ZSGB-BIO, China) was added dropwise and incubated at 37°C for 20 min. Then the secondary antibody was added dropwise and incubated at 37°C for 30 min. After dyeing with DAB, hematoxylin was used for re-dyeing. IHC sections of mouse ovarian tissue were scanned using a digital pathological scanning system (Hamamatsu, Japan). ImageJ V.1.8.0 software was used to analyze the positive expression area of the image, and the data were normalized. Detailed antibody information is listed in Supplementary Table 2.

### IF staining

KGN cells were fixed with 4% paraformaldehyde for 15 min, and then washed with Immunol Staining Wash Buffer (P0106, Beyotime, China). Next, KGN cells were blocked with 10% goat serum at room temperature for 30 min, and incubated with primary antibody at 4°C overnight. The next day, cells were incubated with fluorescently labeled secondary antibodies at 37°C for 1 h, followed by sealing with Antifade Mounting Medium with DAPI (MA0222, Meilunbio, China). Images were taken with a fluorescence inverted microscope (Axio Observer A1, Zeiss, Germany). ImageJ V.1.8.0 software was used to analyze MFI of images and normalize the data. The detailed antibody information is listed in Supplementary Table 2.

### q-PCR

Total RNA was extracted with Trizol, and the concentration and integrity of RNA were determined by spectrophotometry at 260 nm and 280 nm. It was reverse transcribed into cDNA using Evo M-MLV RT Premix for qPCR Kit (AG11706, AG, China) according to the manufacturer’s instructions. The relative mRNA expression levels of Sirt1, p53, and p21 were detected by SYBR Green Premix Pro Taq HS qPCR Kit (AG11718, AG, China). All samples were detected by QuantStudio3 Real-Time PCR System (ABI, USA). The relative expression of RNA was calculated using the 2^−ΔΔCt^ method. Target gene normalization was performed using GAPDH mRNA levels. The sequence of primers is listed in Table 5.

**Table 5:** Sequence of primers for q-PCR.

**Gene**	**Primer**	**Length(bp)**
Sirt1	F: TATACCCAGAACATAGACACGC	204
	R: CTCTGGTTTCATGATAGCAAGC	204
p53	F: TTCCTGAAAACAACGTTCTGTC	85
	R: AACCATTGTTCAATATCGTCCG	85
p21	F: CGTCCAGCGACCTTCCTCATC	109
	R: CCATAGCCTCTACTGCCACCATC	109
GAPDH	F: CAGGAGGCATTGCTGATGAT	138
	R: GAAGGCTGGGGCTCATTT	138

### Western blot analysis

Proteins were extracted from mouse ovarian tissue and KGN cells using RIPA lysis buffer (G2002, Servicebio, China) containing protein phosphatase inhibitor mix (FD1002, Fdbio Science, China). The total protein concentration was measured using BCA protein assay kits (P0012, Beyotime, China). Samples (both cells and tissues) were denatured with 5× loading buffer (FD002, Fdbio Science, China) at 100°C for 5 min. Proteins were separated by SDS-PAGE and transferred to 0.22 μM PVDF membranes (Millipore, USA). It was blocked with 5% nonfat-dried milk for an hour and then incubated with primary antibody at 4°C overnight. The next day, the PVDF membrane was incubated with HRP-conjugated secondary antibody at room temperature for 1 hour. Finally, electrochemiluminescence (ECL) method was used for detection, and imaging was performed using a high-sensitivity imaging analysis system (Bio-Rad, USA). ImageJ V.1.8.0 software was used to analyze the gray value of western blot. According to the protein expression of GAPDH, the protein expression of other genes was normalized. Detailed antibody information is included in Supplementary Table 2.

### Statistical analysis

Experiments were performed at least three times. The data of this study were expressed as mean ± SEM and analyzed using GraphPad Prism 8.0 software. One-way ANOVA was used for comparison between different groups. *p* < 0.05 was considered a statistically significant difference.

## Supplementary Materials

Supplementary Figure 1

Supplementary Tables
