# Supplementary material for: Ningxin-Tongyu-Zishen formula alleviates the senescence of granulosa cells on D-galactose-induced premature ovarian insufficiency mice
Source: Aging (Albany NY). 2024 Feb 28;16(5):4541–62. doi: 10.18632/aging.205607 (PMC10968698; doi:10.18632/aging.205607)
Supplement: Supplementary Figure 1 [file aging-16-205607-s001.pdf]

SUPPLEMENTARY FIGURE

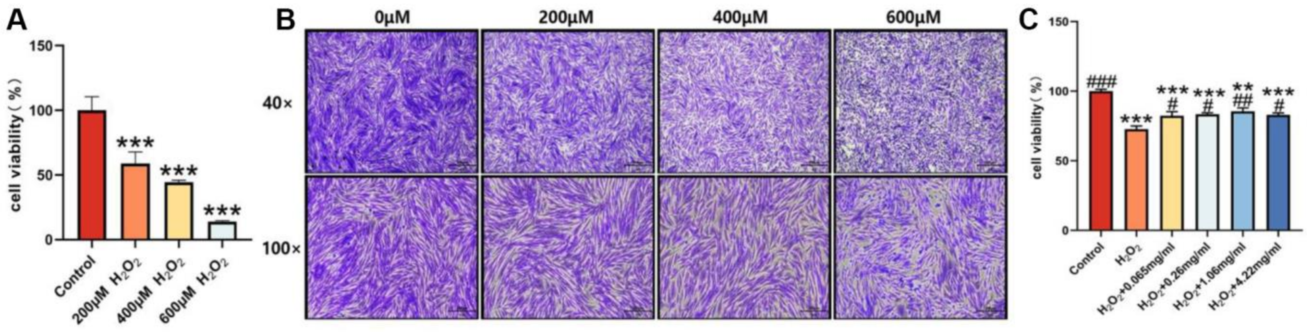

Supplementary Figure 1. The concentration of H_2O_2 and NTZF treated KGN cells was screened. (**A**) CCK-8 was used to detect the effect of different H_2O_2 concentrations on the cell viability of KGN cells. (**B**) Crystal violet staining was used to detect the effect of different H_2O_2 concentrations on the morphology of KGN cells. (**C**) CCK-8 was used to detect the effect of different NTZF concentrations on the cell viability of KGN cells. ($^{*}p < 0.05$, $^{**}p < 0.01$, $^{***}p < 0.001$, versus control group, $n = 3$; $^{\#}p < 0.05$, $^{\#\#}p < 0.01$, $^{\#\#\#}p < 0.001$, versus H_2O_2 group, $n = 3$).
