# Supplementary material for: Ningxin-Tongyu-Zishen formula alleviates the senescence of granulosa cells on D-galactose-induced premature ovarian insufficiency mice
Source: Aging (Albany NY). 2024 Feb 28;16(5):4541–62. doi: 10.18632/aging.205607 (PMC10968698; doi:10.18632/aging.205607)
Supplement: Supplementary Tables [file aging-16-205607-s002.pdf]

SUPPLEMENTARY TABLES

Supplementary Table 1-1. The content of compounds in Chinese medicinals.

No.	Chinese medicinals	Adenosine (ng/mL)	Gallic acid (ng/mL)	Hydroxymethyl-2-furaldehyde+ (ng/mL)	Magnoflorine (ng/mL)	Caffeic acid (ng/mL)	(+)-Catechin (ng/mL)
1	Rehmanniae Radix Praeparata	3086.64	BLQ[a]	34948.03	9.00	2076.73	BLQ
2	Testudinis Carapax et Plastrum	34.51	BLQ	BLQ	11.83	CL[b]	4.05
3	Codonopsis Radix	3857.18	54.15	815.03	13.99	60.16	3.71
4	Cuscutae Semen	1331.49	BLQ	BLQ	69.25	4685.41	5.63
5	Codonopsis Radix	18231.81	122.44	BLQ	2.86	BLQ	8.31
6	Ziziphi Spinosae Semen	1277.19	132.59	BLQ	11568.27	13.39	1810.77
7	Moutan Cortex	3359.06	61548.67	BLQ	1.31	219.79	6317.66
8	Paeoniae Radix Alba	4203.01	35871.30	2489.85	36.05	BLQ	3097.82
9	Dioscoreae Rhizoma	41.09	BLQ	BLQ	0.74	16.30	16.47
10	Bupleuri Radix	2222.11	BLQ	BLQ	1.09	BLQ	17.85

Supplementary Table 1-2. The content of compounds in Chinese medicinals.

No.	Chinese medicinals	Chlorogenic acid (ng/mL)	Paeoniflorin (ng/mL)	Ferulic acid (ng/mL)	Quercetin (ng/mL)	Isorhamnetin (ng/mL)	Linoleic acid (ng/mL)
1	Rehmanniae Radix Praeparata	CL	BLQ	1074.98	CL	CL	—
2	Testudinis Carapax et Plastrum	CL	44.98	BLQ	CL	CL	BLQ
3	Codonopsis Radix	61.24	CL	133.84	CL	CL	BLQ
4	Cuscutae Semen	11638.39	2.16	BLQ	426.01	34.17	612.76
5	Codonopsis Radix	959.44	38.98	5197.23	CL	CL	BLQ
6	Ziziphi Spinosae Semen	CL	BLQ	BLQ	86.23	CL	1424.52
7	Moutan Cortex	CL	44739.84	353.61	194.51	56.26	BLQ
8	Paeoniae Radix Alba	CL	743855.01	BLQ	CL	CL	BLQ
9	Dioscoreae Rhizoma	5.17	2.08	BLQ	CL	CL	687.53
10	Bupleuri Radix	152.24	CL	356.68	299.62	112.34	BLQ

[a]CL indicates the presence of system residue, rendering the value here unreliable, which can be considered as worthless or below the lower limit of quantitation. [b]BLQ indicates to be lower than the lower limit of quantitation.

Supplementary Table 2. The detail information of antibodies.

Antibody	Dilution ratio	Application	Catalog number	Producer details
Sirt1	1:200	IHC	13161-1-AP	Proteintech
p53	1:100	IHC	AF4663	Affinity
PCNA	1:200	IHC	10205-2-AP	Proteintech
Enhanced enzyme-labeled goat anti-mouse/rabbit IgG polymer	–	IHC	PV-9000	ZSGB-BIO
Sirt1	1:100	IF	13161-1-AP	Proteintech
p53	1:50	IF	10442-1-AP	Proteintech
Cy3–conjugated Affinipure Goat Anti-Rabbit IgG(H+L)	1:100	IF	SA00009-2	Proteintech
Sirt1	1:500	WB-tissue	13161-1-AP	Proteintech
p53	1:8000	WB-tissue	10433-1-AP	Proteintech
GAPDH	1:30000	WB-tissue	10494-1-AP	Proteintech
PCNA	1:10000	WB-cell	10205-2-AP	Proteintech
γH2AX	1:5000	WB-cell	ab81299	Abcam
Sirt1	1:3000	WB-cell	13161-1-AP	Proteintech
p53	1:5000	WB-cell	10442-1-AP	Proteintech
p21	1:2000	WB-cell	10355-1-AP	Proteintech
GAPDH	1:20000	WB-cell	10494-1-AP	Proteintech
HRP-conjugated Affinipure Goat Anti-Mouse IgG(H+L)	1:8000	WB-tissue, cell	SA00001-1	Proteintech
HRP-conjugated Affinipure Goat Anti-Rabbit IgG(H+L)	1:5000	WB-tissue, cell	SA00001-2	Proteintech
